# Oxytocin receptor signaling contributes to olfactory avoidance behavior induced by an unpleasant odorant

**DOI:** 10.1242/bio.029140

**Published:** 2018-06-26

**Authors:** Kazumi Osada, Tohru Ohta, Rie Takai, Sadaharu Miyazono, Makoto Kashiwayanagi, Shizu Hidema, Katsuhiko Nishimori

**Affiliations:** 1 Division of Physiology, Department of Oral Biology, School of Dentistry, Health Sciences University of Hokkaido, Ishikari-Tobetsu, Hokkaido, Japan; 2 The Research Institute of Health Science, Health Science University of Hokkaido, Ishikari-Tobetsu, Japan; 3 Department of Sensory Physiology, Asahikawa Medical University, Asahikawa, Hokkaido, Japan; 4 Department of Molecular and Cell Biology, Graduate School of Agricultural Science, Tohoku University, Miyagi, Japan

**Keywords:** Olfactory behavior, Oxytocin receptor, Butyric acid, Avoidance, Y-maze

## Abstract

Oxytocin (OXT) and its receptor (OXTR) regulate reproductive physiology (i.e. parturition and lactation), sociosexual behavior, learned patterns of behavior and olfactory behavior in social contexts. To characterize the function of OXTR in basic olfactory behavior, the present study compared the behavioral responses of homozygous, heterozygous and wild-type mice when these mice were confronted with an unpleasant odorant (butyric acid) in a custom-made Y-maze in the absence of a social context. Wild-type mice avoided the first encounter with the butyric acid odorant, whereas homozygous and heterozygous mice did not. However, both heterozygous and wild-type mice habituated when confronted with the butyric odorant again on the following 2 days. By contrast, homozygous mice failed to habituate and instead avoided the location of the odorant for at least 3 days. These data suggest that homozygous and heterozygous mice display abnormal olfactory responses to the presentation of an unpleasant odorant. Our studies demonstrate that OXTR plays a critical role in regulating olfactory behavior in the absence of a social context.

## Abstract (precis)

**Summary:** Homozygous mice exhibited abnormal olfactory behaviors, namely failure in the acute avoidance of butyric acid and in habituation behavior, in the absence of a social context.

## INTRODUCTION

Oxytocin (OXT) is a nonapeptide hormone synthesized in the paraventricular and supraoptic nuclei of the hypothalamus (Gainer, 1998; Gainer and Wray, 1992) and is secreted from the posterior pituitary gland. In addition to its classic effects on the release and ejection of milk, OXT plays important roles in social behavior. OXT receptor (OXTR)-deficient mice express abnormal sexual and maternal behaviors, affiliations, and social memory, and show increased aggression (Takayanagi et al., 2005). OXTR null mice also demonstrate resistance to changing a learned pattern of behavior (Sala et al., 2011) and have been used as a model of autism spectrum disorder (ASD) (Insel, 2010).

Moreover, recent reports indicate that OXT modulates context-dependent olfactory behavior (Choe et al., 2015; Oettl et al., 2016). Mice with a conditional OXTR gene deletion, selective to the anterior olfactory nucleus, show a deficit in recognition of same-sex conspecifics using olfaction, although these mice perform object and non-social odor recognition at levels comparable to control mice (Oettl et al., 2016). In addition, OXTR signaling is crucial for the synchronization of odors to social cues in rats but is not important for synchronization of non-social cues (Choe et al., 2015). Moreover, Lee et al. (2008) tested a non-social odor in a non-social context using both male and female mice with forebrain oxytocin receptor knockout and total body oxytocin receptor knockout using a habituation-dishabituation test with the odor of almond extract as the non-social odorant. There were no sex or genotypic differences in the response of these mice to the odor of almond extract. Thus, the results of these previous studies suggest that OXTR is dispensable for odor detection in the absence of a social context. However, for several reasons, we hypothesized that olfactory disorders might occur in heterozygous and homozygous mice in a non-social context.

First, previous neurophysiological studies have revealed that OXT fibers project to various brain regions (Gimpl and Fahrenholz, 2001) wherein OXT functions as a neurotransmitter or neuromodulator. The OXTR is abundantly present in several brain regions, including some cortical areas, the olfactory system, the basal ganglia, the limbic system, the thalamus, the hypothalamus, the brainstem, and the spinal cord (Gimpl and Fahrenholz, 2001; Vaccari et al., 1998; Yoshimura et al., 1993), suggesting that OXTRs in the central nervous system have a wide variety of effects, including olfaction. Especially the granular cell layer of the main olfactory bulb, which is a region with a particularly high density of OXTRs (Yoshida et al., 2009; Yu et al., 1996). Therefore, it is conceivable that homozygous mice may be predisposed to olfactory disorders that are not associated with a social context setting. Second, OXTR is also abundantly distributed to brain centers that are key players in the process of emotion, emotional behavior and fear behavior (Guzmán et al., 2013; Kashiwayanagi et al., 2017), namely, lateral septal nucleus, central amygdaloid nucleus, cortical and basomedial amygdaloid nucleus and ventromedial hypothalamic nucleus (Yoshida et al., 2009; Yu et al., 1996), suggesting that the behavioral response released by pleasant and/or unpleasant odor may be controlled by OXTR in a non-social context. Third, as mentioned in the Diagnostic and Statistical Manual of Mental Disorders 5th edition (American Psychiatric Association, 2013), People with ASD are often characterized by atypical sensory behavior, namely, hyper- or hypo-reactivity to sensory input including olfaction. However, no previous studies have reported olfactory disorders in Oxtr-deficient mice in a non-social context.

Thus, the present study aimed to determine whether abnormal changes in olfactory behavior occur in homozygous mice within a non-social context. We used butyric acid, a typical unpleasant odorant emitted from spoiled food, and conducted a set of behavioral tests in these mice. Our results demonstrated reliable abnormalities in simple avoidance olfactory behavior in the absence of a social context in homozygous mice.

## RESULTS

### Open-field test

**Fig. 1.**
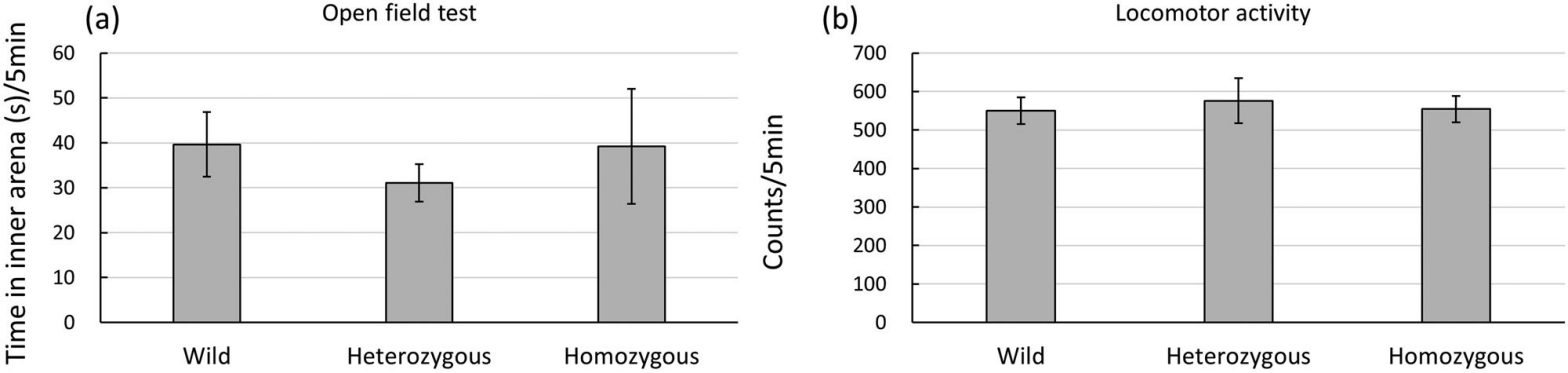
**Homozygous (OXTR-deficient) and heterozygous mice show normal anxiety-like behavior and locomotion.** Anxiety (A) was measured by determining the amount of time spent in the central area of an open-field apparatus for up to 5 min. (B) Locomotor activity was measured as a signal produced by the movement of mice on an Animex activity meter within 5 min.

As shown in Fig. 1A and B, no significant differences in anxiety behavior as measured using the open-field test and an Animex activity meter were detected among the three mouse groups. The data from each group were analyzed by two factor ANOVA with genotype of Oxtr and sex as independent factors. This analysis did not reveal a significant main effect of the genotype of Oxtr [*F* (2, 39)=1.001, *P*=0.377] or of sex [*F* (1, 39)=1.975, *P*=0.168] in the open field test. The mean locomotor activity scores using the Animex activity meter were also not significantly different between the Oxtr genotypes [*F* (2, 39)=0.756, *P*=0.477] or between sexes [*F* (2, 39)=1.555, *P*=0.221]. These data indicated that neither the expression level of OXTR nor the sex difference affected anxiety-like behavior of mice as measured by the time spent in the center area of an open-field apparatus and by locomotor activity determined using an Animex activity meter.

### Avoidance of butyric acid in the Y-maze

**Fig. 2.**
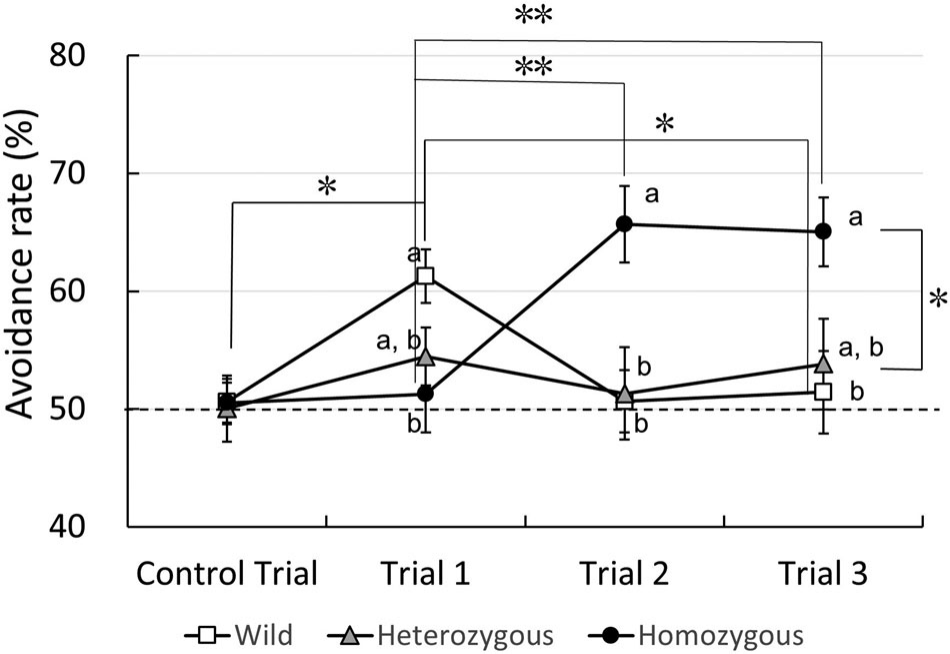
**Changes in the results of the avoidance test over time.** Data from homozygous (closed circle), heterozygous (gray triangle), and wild-type (open square) mice. To show the avoidance rate correctly, the Y-axis shows values between 40–70%. The statistical significance of the differences in the avoidance rates of the three groups was assessed using two factors repeated-measure ANOVA followed by Bonferroni post-hoc test (*P<0.05, **P<0.01), different letters between the groups indicate a significant difference (*P*<0.05).

**Fig. 3.**
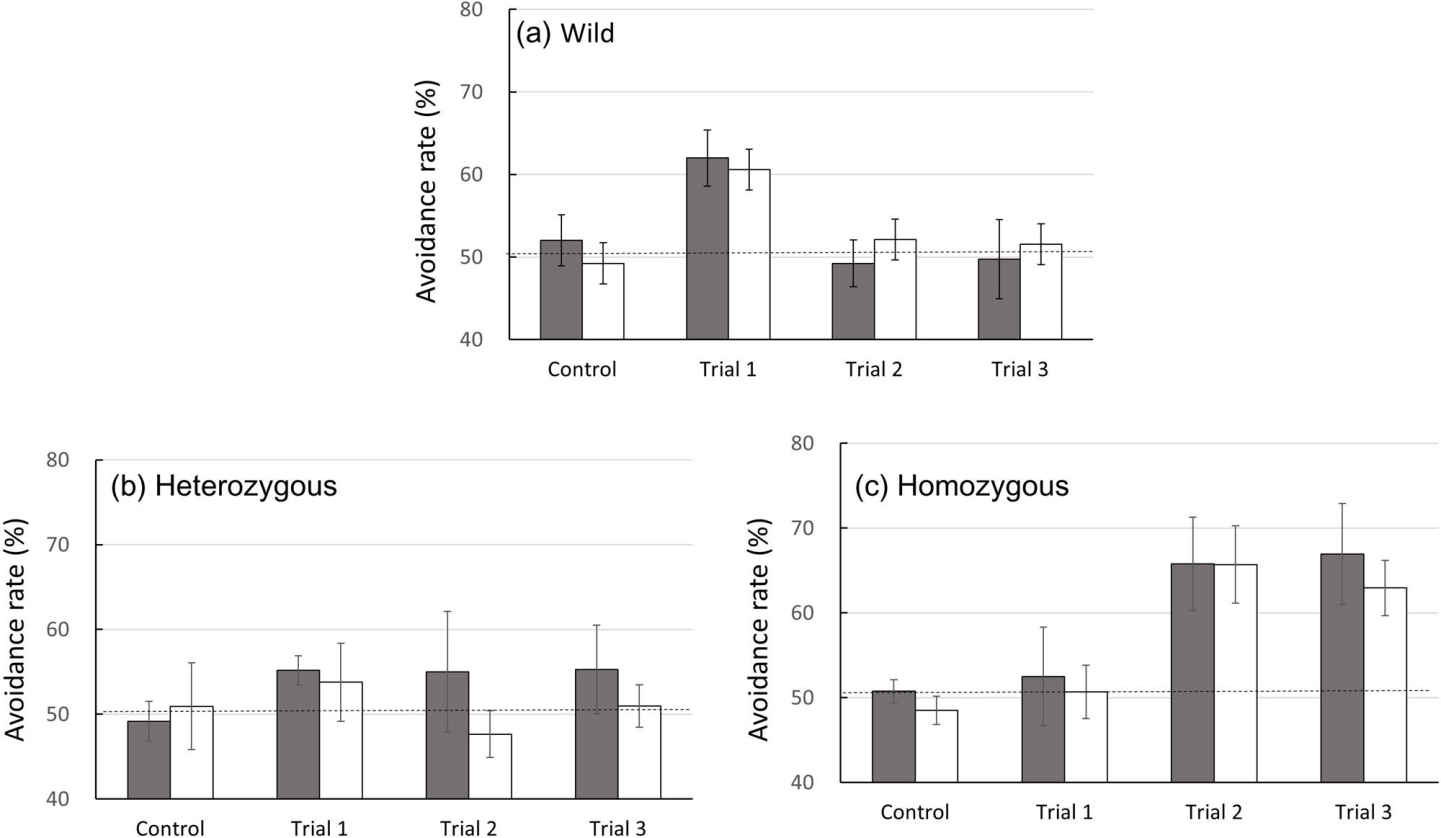
**Comparison of time changes in avoidance rates between males and females.** Data for (A) wild-type, (B) heterozygous and (C) homozygous mice are presented. The Y-axis shows values between 40–70%. The statistical significances of the differences between the avoidance rates of males (gray column) and females (open column) were assessed using repeated-measure ANOVA followed by Bonferroni post-hoc test.

To determine whether homozygous mice avoid unpleasant odors in non-social and non-context conditions, the Y-maze avoidance test was conducted. The total remaining times in the short arms of the Y-maze between the three mice groups were almost the same (wild; control trial, 106.8±14.7 s, average of trials 1∼3, 111.6±12.4 s, heterozygous; control trial, 111.4±11.2 s, average of trials 1∼3, 106.9±11.8 s, Oxtr-deficiency; control trial, 121.3±13.2 s, average of trials 1∼3, 112.4±14.2 s). However, some avoidance rates were different between the mice groups. Two factor repeated-measure ANOVA revealed a main effect between Oxtr genotypes [*F* (4.985, 104.69)=4.769, *P*=0.001] but not between sexes [*F* (2.570, 129)=0.007, *P*=0.998]. Bonferroni test indicated that the avoidance activity of homozygous mice toward butyric acid was significantly higher than that of heterozygous (*P*=0.040) mice (Fig. 2). There were no differences between sexes in each genotype [wild; *P*=0.974, homozygous; *P*=0.235, heterozygous; *P*=0.279, Bonferroni test] (Fig. 3A–C). Therefore, we compared mouse genotype groups using the total male and female data.

For the wild-type group, there were significant difference between the control trial and trial 1 (50.63±2.00% vs 61.29±2.34%; *P*=0.016) or between trial 1 and trial 3 (61.29±2.34% vs 50.66±3.40%; *P*=0.024: Bonferroni test). For the homozygous mice group, there were significant differences between trial 1 and trial 2 (49.72±1.70% vs 65.73±3.26%; *P*=0.006), and trial 3 (64.61±3.01% *P*=0.007: Bonferroni test). For the heterozygous group, there were no significant differences between trials (Fig. 2).

In trial 1, mice in the wild-type group, but not in the heterozygous or homozygous groups, showed statistically significant avoidance of butyric acid. Bonferroni test showed significant differences between the wild-type (61.29%) and homozygous groups (51.28%; *P*=0.019; Fig. 2). Moreover, when the avoidance rates between the control trial group and trial 1 group were compared, wild-type mice significantly avoided the butyric acid odorant (control, 50.63±2.00%; trial 1, 61.29±2.34%; *P*=0.001, paired *t*-test) but not the other groups (control, 50.04±1.51%; trial 1, 54.47±2.46%; *P*=0.210; for heterozygous, control, 50.55±2.60%; trial 1, 51.28±3.39%; *P*=0.856; for homozygous, paired *t*-test; Fig. 3). The results of both statistical analysis clearly indicated that wild-type mice avoided butyric acid on first encounter, but the other mice groups did not.

In trial 2, wild-type mice appeared to become habituated to the unpleasant odor of butyric acid [50.63±2.00% (control trial) vs 50.68±2.64% (trial 2); *P*=1.000: Bonferroni test], whereas homozygous mice failed to become habituated to the odorant and significantly avoided it. Bonferroni post hoc test showed significant differences between the wild-type and homozygous groups (*P*=0.006), and between the heterozygous and homozygous groups (*P*=0.009; Fig. 2). In trial 3, Bonferroni test showed significant differences between the wild-type and homozygous groups (*P*=0.024), but not between the heterozygous and homozygous groups (*P*=0.090; Fig. 2).

## DISCUSSION

Our results demonstrated that OXTR deficiency in mice is associated with an abnormal olfactory context. Using a set of olfactory behavioral trials, we observed two different types of abnormal olfactory behavior between homozygous and/or heterozygous mice and wild-type mice.

**Fig. 4.**
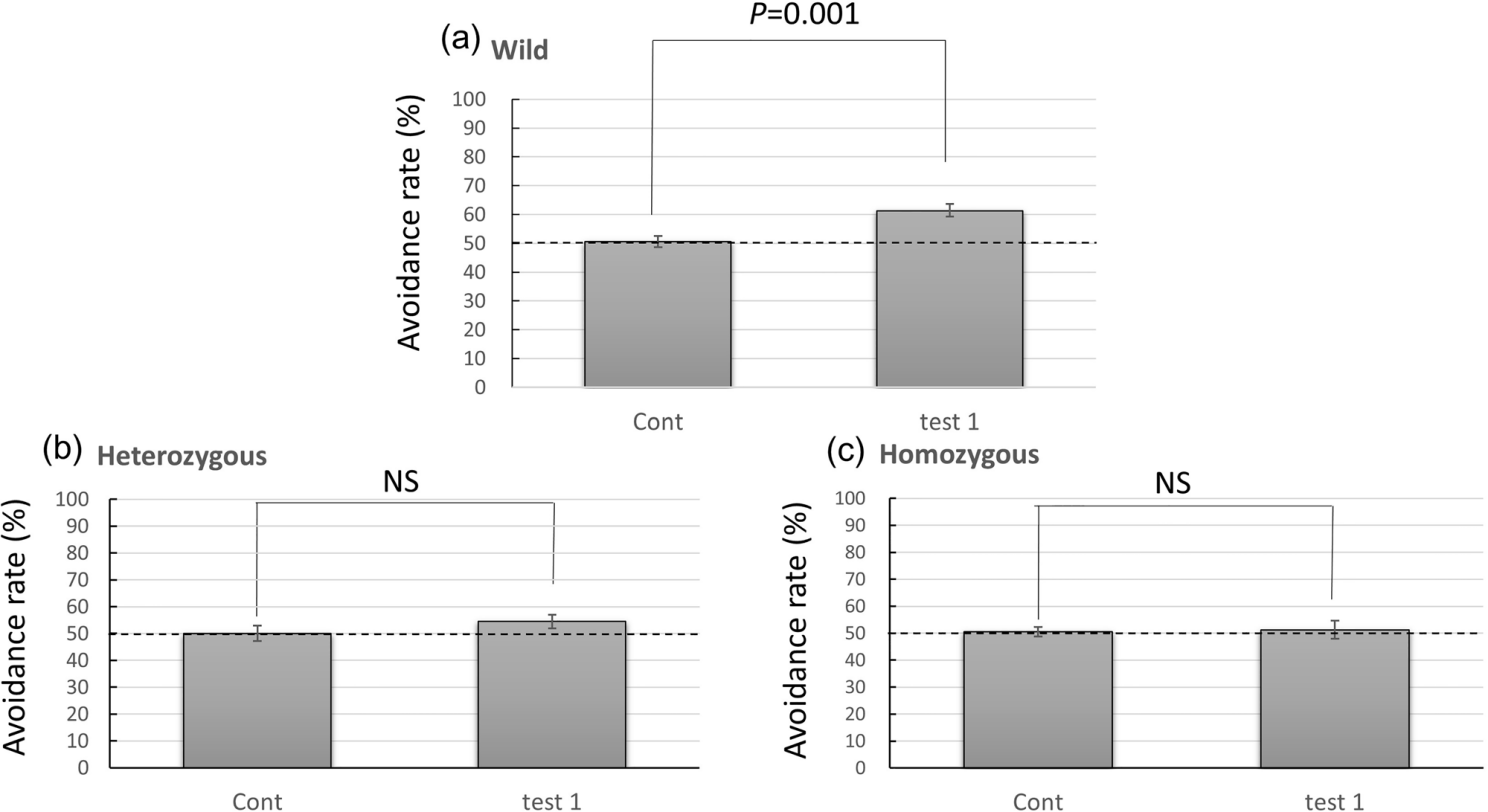
**Comparison between the control trial and trial 1.** Data for (A) wild-type, (B) heterozygous and (C) homozygous mice are presented. The rate at which mice avoided the unpleasant butyric acid odorant in the Y-maze, that is, the avoidance rate, of trial 1 was taken to be the amount of time spent in the short arm containing the control odorant divided by the total amount of time spent in both short arms (control versus butyric acid), multiplied by 100. Water was substituted for butyric acid in control experiments (control versus control). Values are the mean±s.e.m. of at least eight animals in each group. The statistical significance of the differences between the avoidance rates was assessed using the paired *t*-test. Wild type, *P*=0.001; heterozygous, not significant (NS); homozygous, NS.

First, when test mice were confronted with butyric acid, homozygous and heterozygous mice failed to avoid this odorant, whereas wild-type mice significantly avoided it (Figs 2 and 4A­–C). Previous studies have indicated that 5–10 μl butyric acid induces modest but reliable avoidance in wild-type mice (Utsugi et al., 2014) and rats (Endres and Fendt, 2009). To the best of our knowledge, the failure of homozygous and heterozygous mice to show an avoidance response to an unpleasant and/or novel odor (butyric acid) is a novel finding of abnormal behavior in these mice.

The OXTR null mouse reportedly displays specific deficits in social behaviors, namely, sociability and social novelty (Sala et al., 2011). It is a matter of controversy how these mice acquire social information from conspecific animals; however, in animals that rely on chemical communication for the regulation of social and sexual interactions, there is some evidence that animal social behavior is determined by the odors of the animals. In addition, both homozygous and heterozygous mice exhibited these social behavior deficits (Sala et al., 2013). In the present study, we similarly found that both homozygous and heterozygous mice failed to avoid the butyric acid odorant in their first encounter (trial 1). Therefore, it is conceivable that the mechanism underlying the hypo-reactivity of homozygous and heterozygous mice to butyric acid at first presentation is the same as that underpinning the induction of the social behavioral deficit observed in these mice.

Second, in addition to the dysfunction in their acute response to the unpleasant odorant in trial 1, homozygous, but not heterozygous or wild-type mice, displayed persistent avoidance responses to butyric acid odorant as demonstrated during trials 2 and 3 (Fig. 2). One previous study demonstrated that OXTR null mice show a phenotype that is resistant to changing a learned pattern of food selection behavior evaluated using a T-maze (Sala et al., 2011). Such inflexible behavior has only been observed for OXTR null (homozygous) mice, not for heterozygous or wild-type mice (Sala et al., 2013), and persistent hyper-reactivity of homozygous mice was observed in trials 2 and 3 in the present study. Therefore, abnormal food selection behavior may be attributable to an abnormality in the inflexible olfactory behavior observed in the present study.

In addition to abnormal social symptoms (Leekam et al., 2007), hypo-reactivity to sensory stimuli including odors (Rogers et al., 2003; Legiša et al., 2013) has been observed as a typical trait of people with ASD (Kumazaki et al., 2016; Legiša et al., 2013; Wicker et al., 2016). Moreover, inflexible behavior and hyper-reactivity to odors have also been observed in people with ASD. For example, they judge odors to be more intense than normal people (Rogers et al., 2003; Wicker et al., 2016). In addition, abnormal behaviors, such as excessive smelling of an object, and extreme reactions to the smell of food are also observed (American Psychiatric Association, 2013). The behavioral data in trial 2 and 3 suggests that the homozygous mice may be hyper-reactive and inflexible in their response to olfactory input, as observed in people of ASD. Together, the behavioral data presented in this study indicates that the homozygous mice, and in part heterozygous mice, have sensory disorders in olfaction similar to those of people with ASD.

In contrast, OXTR deficiency did not induce anxiety-like behavior or alter motor activity in mice (Fig. 1). Previous studies (Curley et al., 2012; Pagani et al., 2015; Sala et al., 2011) indicate that OXTR null mice display motor activity and anxiety behavior similar to normal mice, consistent with our results. Together, the present results indicate that the abnormal olfactory behavior of the homozygous and heterozygous mice was not due to anxiety and/or hyperactivity in the absence of olfactory input.

It is a matter of debate how oxytocin modulates function in the central nervous system and induces olfactory behaviors. One previous report (Reed et al., 2013) demonstrated that the butyric acid odorant evokes robust brain blood oxygen level-dependent contrast imaging activation across olfactory, sensory, memory, and limbic regions in rats. Blockade of OXTRs modulates odor-evoked neural activity, particularly in the amygdala. The loss of the Oxtr gene in the central nucleus of amygdala (Pagani et al., 2011), and the lateral septum (Guzmán et al., 2013) impairs avoidance and fear, indicating that the part of the limbic system that induces emotional behavior may be the target of the modulating function of oxytocin (Sobota et al., 2015; Waldherr and Neumann, 2007). In addition, the pyriform cortex (Choe et al., 2015) and the anterior olfactory nucleus (Oettl et al., 2016) are also potential centers of olfactory behavior in social contexts. Moreover, the most abundant expression of the Oxtr gene is observed in the main olfactory bulb, the primary center of olfaction (Yoshida et al., 2009). These studies indicate that the function of these brain regions, which transduce and analyze olfactory information, are controlled by oxytocin. Clearly, further study will be required to explore which ‘brain area(s)’ or combinations thereof are involved in the olfactory dysfunctions observed in the present study. Finally, although we demonstrated simple behaviors of mice in an experimental setting, our results have expanded the phenotype of homozygous and heterozygous mice by adding two different types of changes in olfactory behaviors to unpleasant/novel odor in the absence of a social context.

## MATERIALS AND METHODS

### Experimental animals

Mice were cared for in accordance with the National Institute of Health's ‘Guide for the Care and Use of Laboratory Animals’. The Animal Ethics and Research Committee of the Health Sciences University of Hokkaido approved the experimental protocols prior to the initiation of the study (approval ID, 059).

Oxtr-Venus knockin (homozygous) , Oxtr^+/+^ (wild-type), and Oxtr^+/v^ (heterozygous) mice generated by Yoshida et al. (2009) were bred and used in the present study. These mice were maintained on a mixed 129×C57BL/6J genetic background. In the behavior tests, we used homozygous, wild-type, and heterozygous mice from heterozygous intercrosses. Behavioral experiments were performed using sexually inexperienced 3–5-month-old mice [wild type, *n*=16, (8 male, 8 female); heterozygous, *n*=16 (8 male, 8 female); homozygous, *n*=13, (5 male, 8 female)]. The animals were housed in groups of two to three animals in polycarbonate cages until the start of the behavioral experiments in a room maintained at 22°C with a photoperiod of 12 h (non-reversed 12 h light/dark cycle) in a sterile animal facility and provided with *ad libitum* access to water and a standard murine diet (Lab Chow, MF, Oriental Yeast, Tokyo, Japan).

### Open-field test

Open-field activity was measured using a minor modification of the method described previously (Rich et al., 2014). Mice were brought into the room where the behavioral tests were conducted 60 min prior to the test start. Mice were placed in the center of the open-field apparatus (rectangle polycarbonate, 42×28 cm) illuminated at approximately 200 lux and allowed to explore for 5 min (Harro et al., 1999; Shang et al., 2017). The animals were tracked to determine the amount of time spent in the center area (28×14 cm). Recording and analysis was conducted with a video camera (Ivis HF R42, Canon, Tokyo, Japan). Locomotor activity was measured at the same time and quantified using an Animex activity meter for 5 min. For quantification, the mice were placed on top of the meter, and each movement produced a signal caused by variations in the inductance and capacity of the resonance circuit of the apparatus. The sensitivity of the activity meter was adjusted to record mainly locomotion. These signals were automatically converted to numbers.

### Y-maze avoidance test

Butyric acid was used to stimulate avoidance in each group of the three groups of mice. The mice were examined only in one set of avoidance tests, which were conducted in a blinded manner using a custom-made Y-maze (long arm length: 450 mm, short arm length: 400 mm, arm width: 100 mm) (Osada et al., 2011; Yamaguchi et al., 1981). Before initiation of the experiment, mice were habituated to the Y-maze for 4 min. On the first experimental day, filter papers of the same size (2.5×2.5 cm) spotted with water (10 µl) to serve as controls were simultaneously inserted into both of the short arms of the Y-maze. The mouse was placed on the end of the long arm. After the gate in front of the mouse was opened, the amount of time the mouse spent in both short arms was measured for 4 min (control trial). On the next day, 10 µl (56 μ mol) butyric acid aqueous solution was applied to the filter paper (2.5×2.5 cm), which was placed on the end of one of the short arms of the Y-maze; filter paper spotted with water was inserted into the other short arm. Butyric acid was presented randomly in each arm in each trial. Animals were then placed on the long arm of the Y-maze as before, and the amount of time the mouse spent in both short arms was measured for 4 min (trial 1). This same protocol was repeated the next day (trial 2) and the day after that (trial 3).

Odor sources were inserted at random into either short arm. Each trial was conducted between 12:00 (noon) and 17:00 h. The floor of the test area was replaced with clean bench paper between each trial to eliminate residual odorant cues. The avoidance rate was taken to be the amount of time spent in the short arm containing the control odor divided by the total amount of time spent in both short arms (control versus butyric acid), multiplied by 100.

### Statistical analysis

Data are presented as means±s.e.m. The statistical significance of the differences between groups and between sexes was assessed using an analysis of variance (ANOVA), or assessed using a repeated-measure ANOVA when appropriate, with the Bonferroni post hoc test. The statistical significance of the differences between the results of the control session and trial 1 session in each of the mouse groups was assessed using the paired *t*-test.
